# Supplementary figures and images for: A rotifer-derived paralytic compound prevents transmission of schistosomiasis to a mammalian host
Source: PLoS Biol. 2019 Oct 17;17(10):e3000485. doi: 10.1371/journal.pbio.3000485 (PMC6797223; doi:10.1371/journal.pbio.3000485)

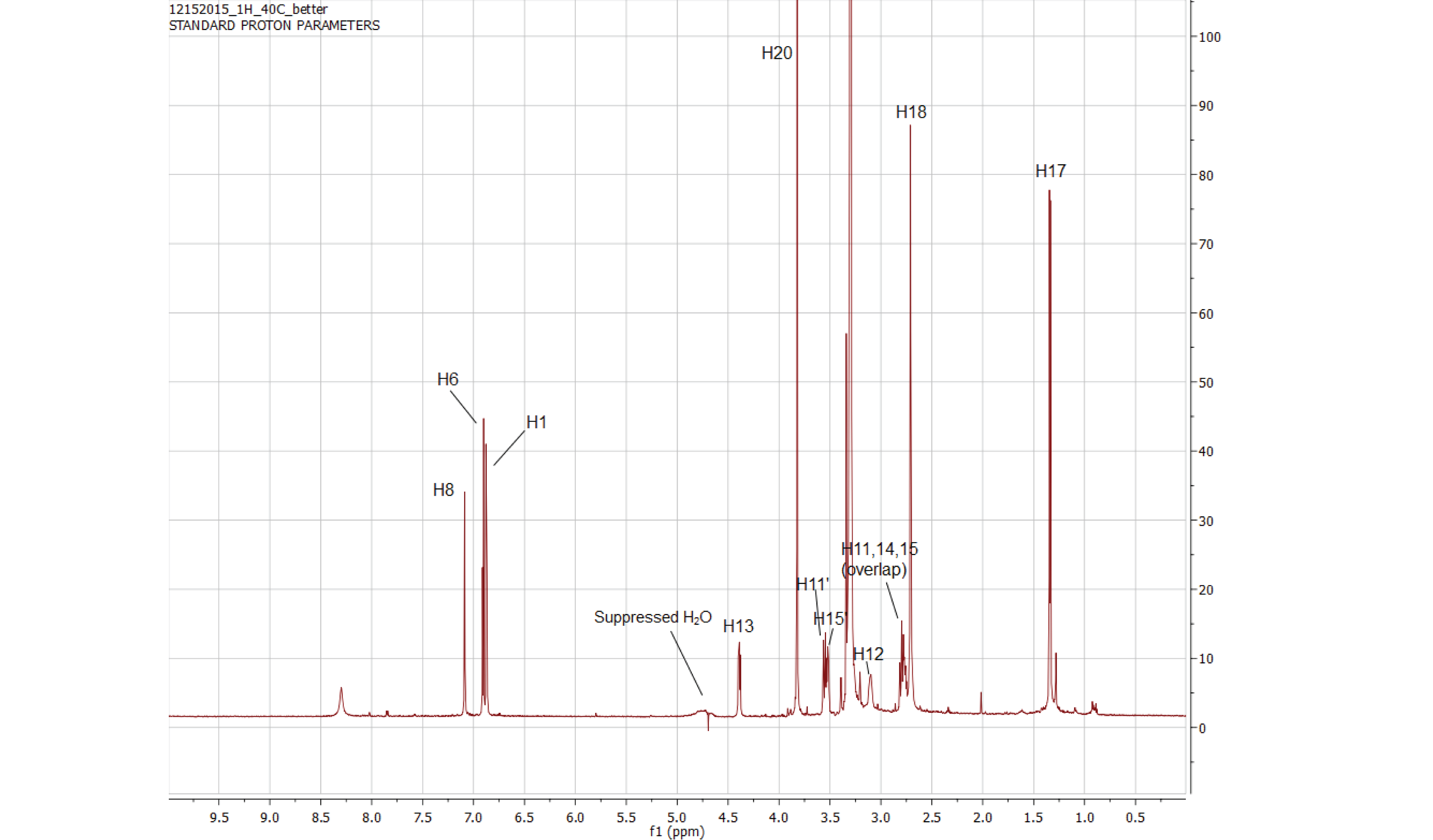

Supplement: S1 Fig — Peak areas of the nonoverlapping peaks were integrated and protons (δH 1.34, 3.10, 3.52, 3.56, 3.81, 4.40, 6.86, 6.90, and 7.09) showed integer ratios, supporting the mass spectrometry results that their signals were from the same compound. After adding the integration of overlapping peaks (δH 2.70, 2.72, 2.77, 2.79), a total of 19 protons were discovered, consistent with the best-fitting formula from the mass spectrometry results: (C16H20N2O2). NMR, nuclear magnetic resonance; SPF, Schistosome Paralysis Factor. (TIF) [file pbio.3000485.s001.tif]

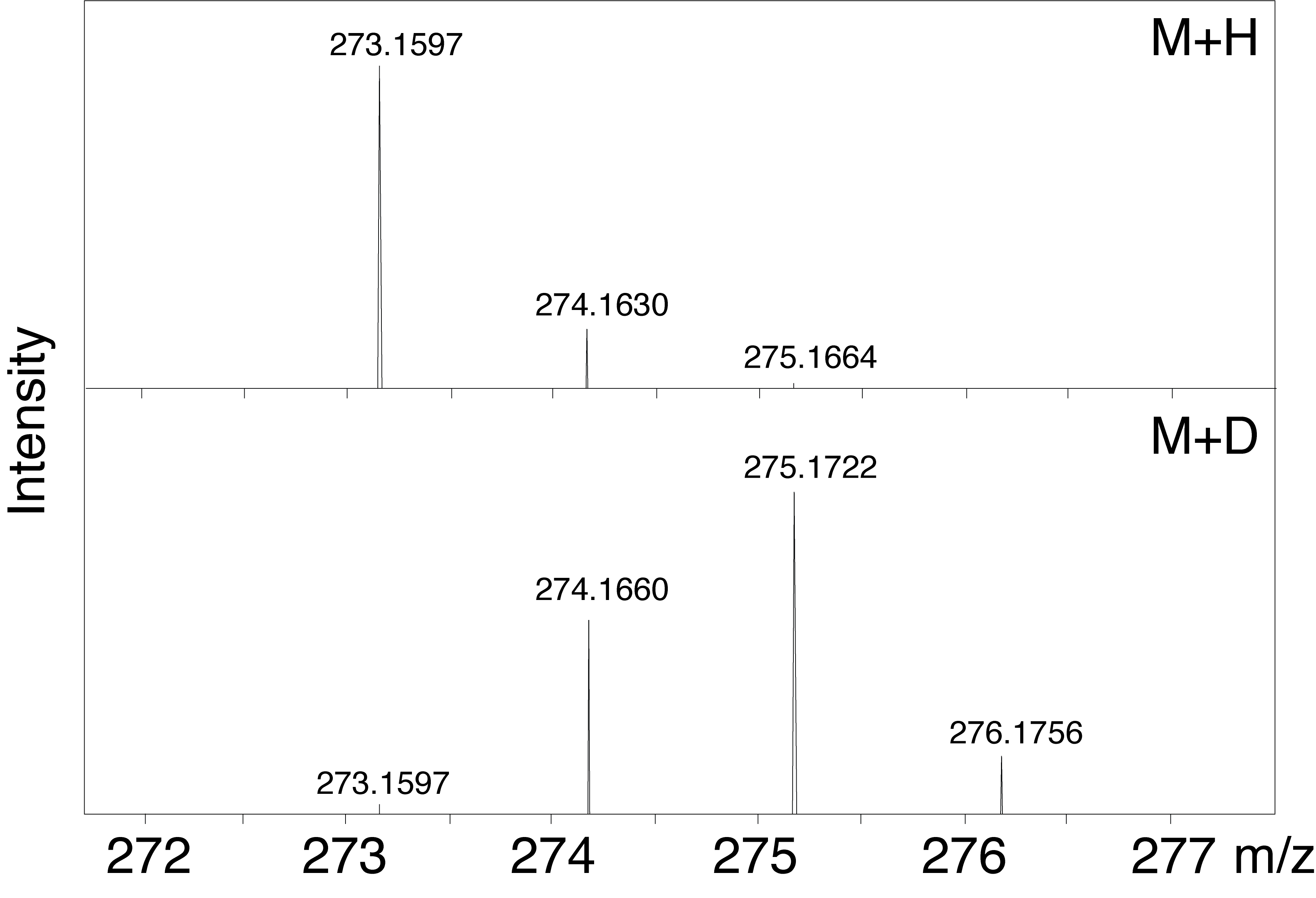

Supplement: S2 Fig — Before deuterium exchange (top panel), 273.1597 was the measured m/z of the target molecule. After deuterium exchange (bottom panel), m/z of the base peak increased to 275.1722 (deuterium singly charged target molecule with one proton replaced by deuterium), suggesting the presence of one exchangeable proton in SPF. FTMS, Fourier-transform mass spectrometry; SPF, Schistosome Paralysis Factor. (TIF) [file pbio.3000485.s002.tif]

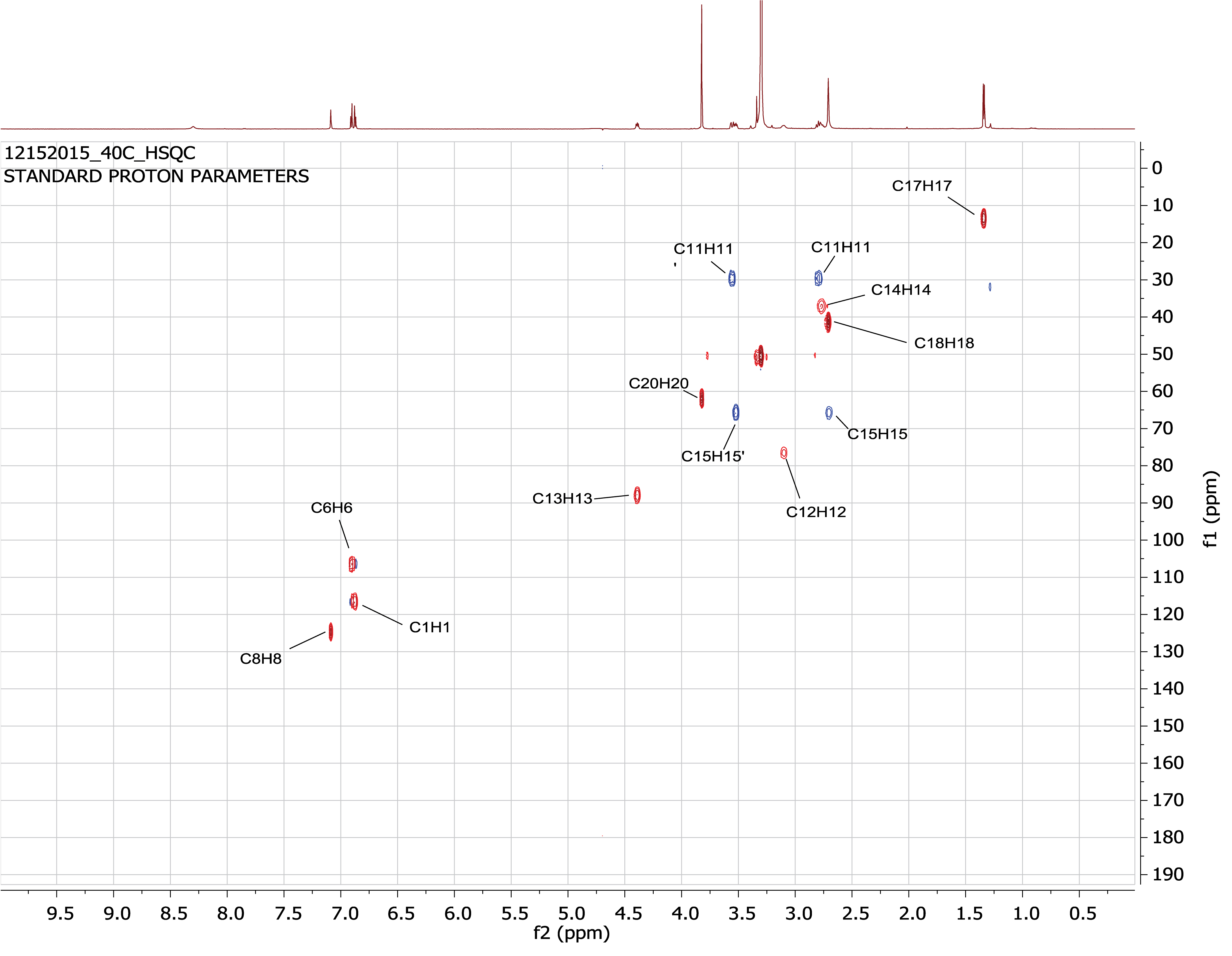

Supplement: S3 Fig — HSQC revealed the cross-correlation between directly bonded proton and carbon nuclei and determined the number of methyl, methylene, and methine groups. A total of 19 protons were attached to 11 carbons, including 3 methyl groups (δC 14.0, δH 1.34; δC 41.7, δH 2.71; δC 62.2, δH 3.81), 2 methylene groups (δC 29.0, δH 2.79, 3.56; δC 65.8, δH 2.70, 3.52), and 6 methine groups (δC 116.8, δH 6.86; δC 106.6, δH 6.90; δC 124.9, δH 7.09; δC 76.7, δH 3.10; δC 88.2, δH 4.40; δC 37.2, δH 2.77). The other 5 carbons that did not show up in the HSQC spectrum are the quaternary carbons. Based on the carbon chemical shift, the 2 methyl groups (δC 41.7, δH 2.71, and δC 62.2, δH 3.81) are likely to be bound to nitrogen and oxygen, respectively. HSQC, heteronuclear single quantum coherence spectroscopy; NMR, nuclear magnetic resonance; SPF, Schistosome Paralysis Factor. (TIF) [file pbio.3000485.s003.tif]

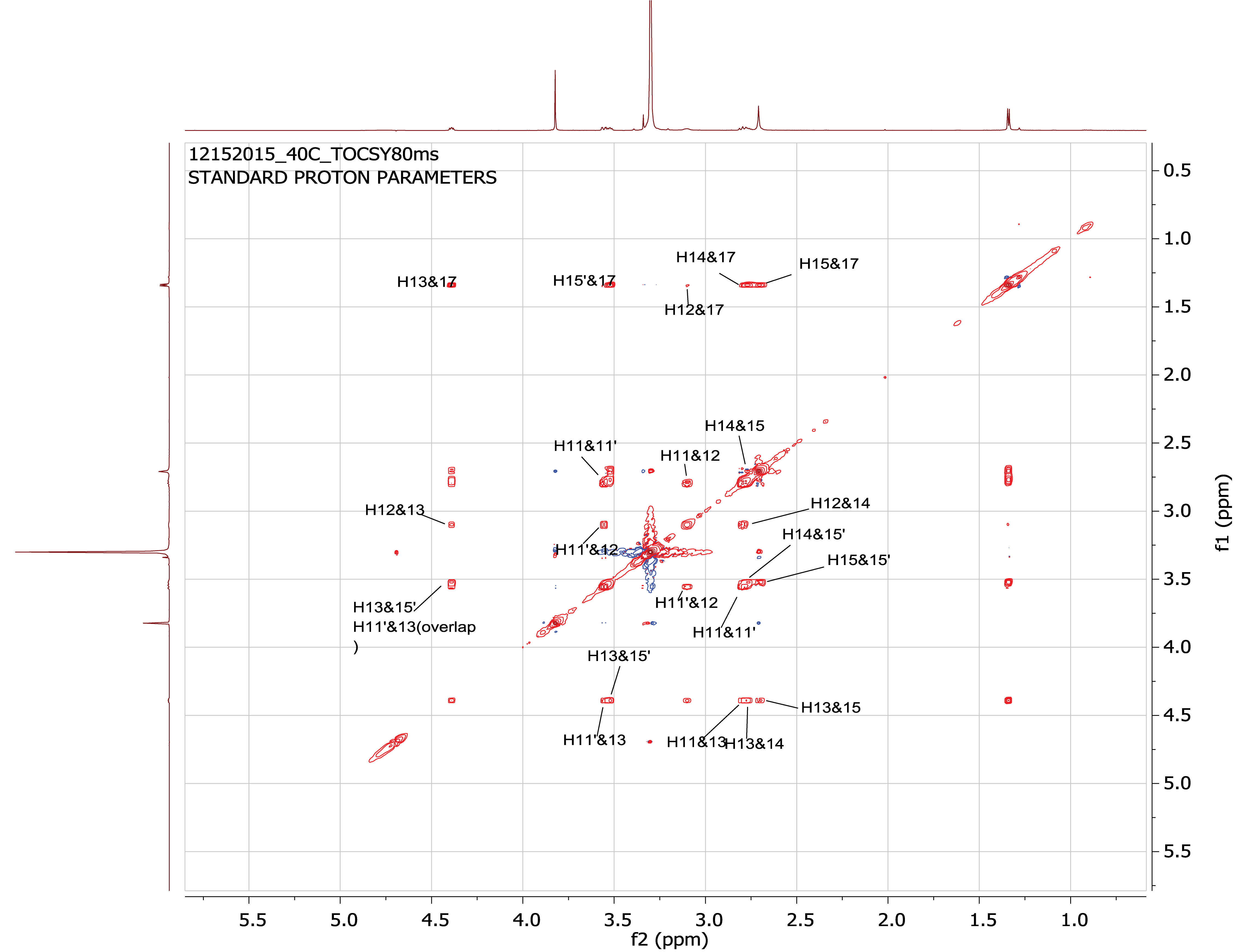

Supplement: S4 Fig — TOCSY revealed that the aliphatic protons except the 2 methyl groups (δH 2.71 and 3.81) found binding to N and O in HSQC (S3 Fig) are from a single spin system. Cross-peaks were also observed among the aromatic proton δH 7.09 and the aliphatic protons (δH 3.56, 2.79 and 3.10) due to long-range couplings. HSQC, heteronuclear single quantum coherence spectroscopy; NMR, nuclear magnetic resonance; SPF, Schistosome Paralysis Factor; TOCSY, total correlation spectrometry. (TIF) [file pbio.3000485.s004.tif]

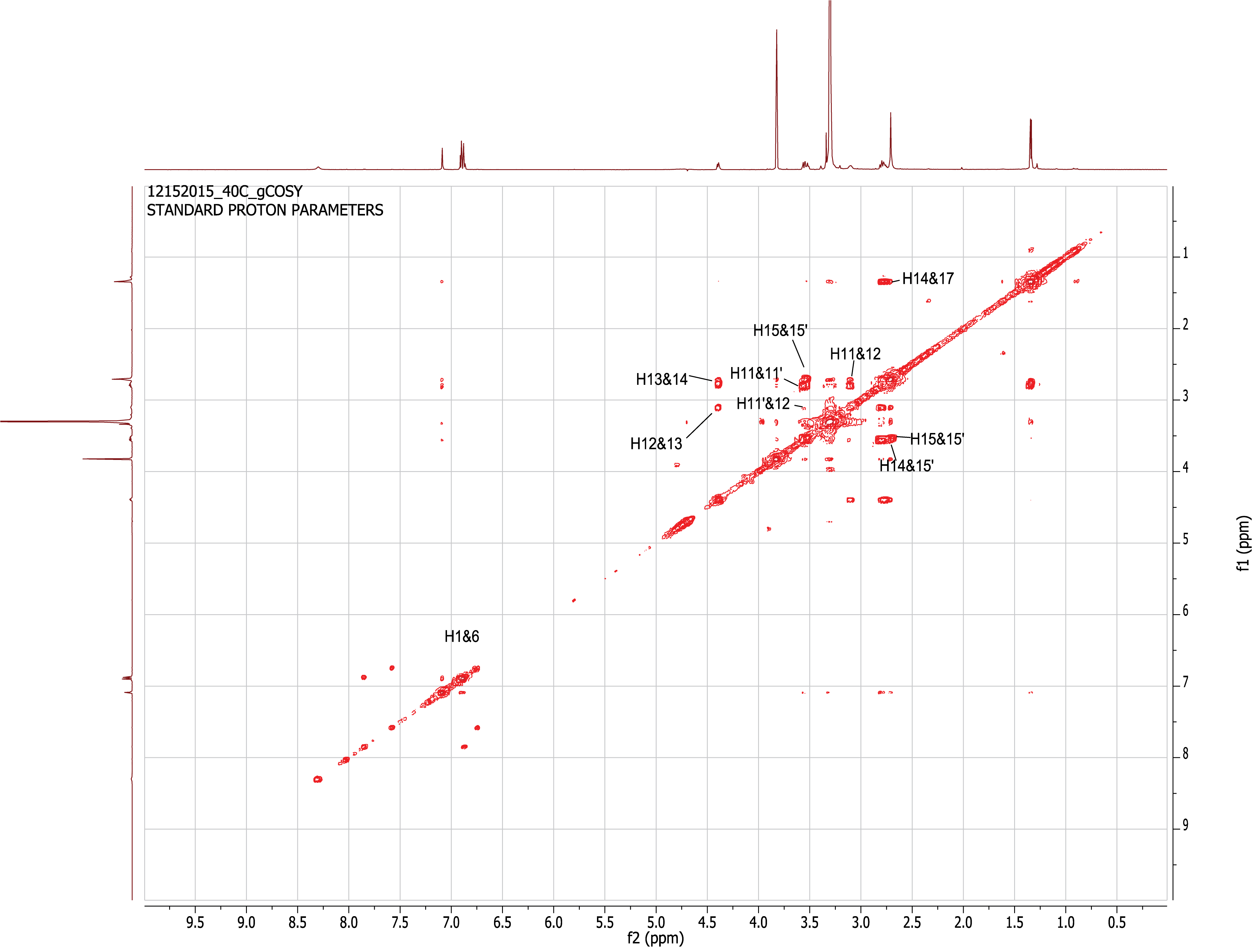

Supplement: S5 Fig — Based on HSQC, protons 11 and 11ʹ (δH 2.79 and 3.56) are on the same carbon. Both have cross-peaks with proton 12 (δH 3.10) on COSY, which has an additional cross-peak with proton 13 (δH 4.40). This suggests CH2 (C11, H11, and 11ʹ)-CH (C12, H12)-CH (C13, H13) connectivity. Similarly, proton 14 (δH 2.77) is connected to CH (C13, H13). Methyl group CH3 (proton 17, δH 1.34) and CH2 group (proton 15, 15ʹ, δH 2.70, 3.52) are directly connected to CH (proton 14). COSY, correlation spectroscopy; HSQC, heteronuclear single quantum coherence spectroscopy; NMR, nuclear magnetic resonance; SPF, Schistosome Paralysis Factor. (TIF) [file pbio.3000485.s005.tif]

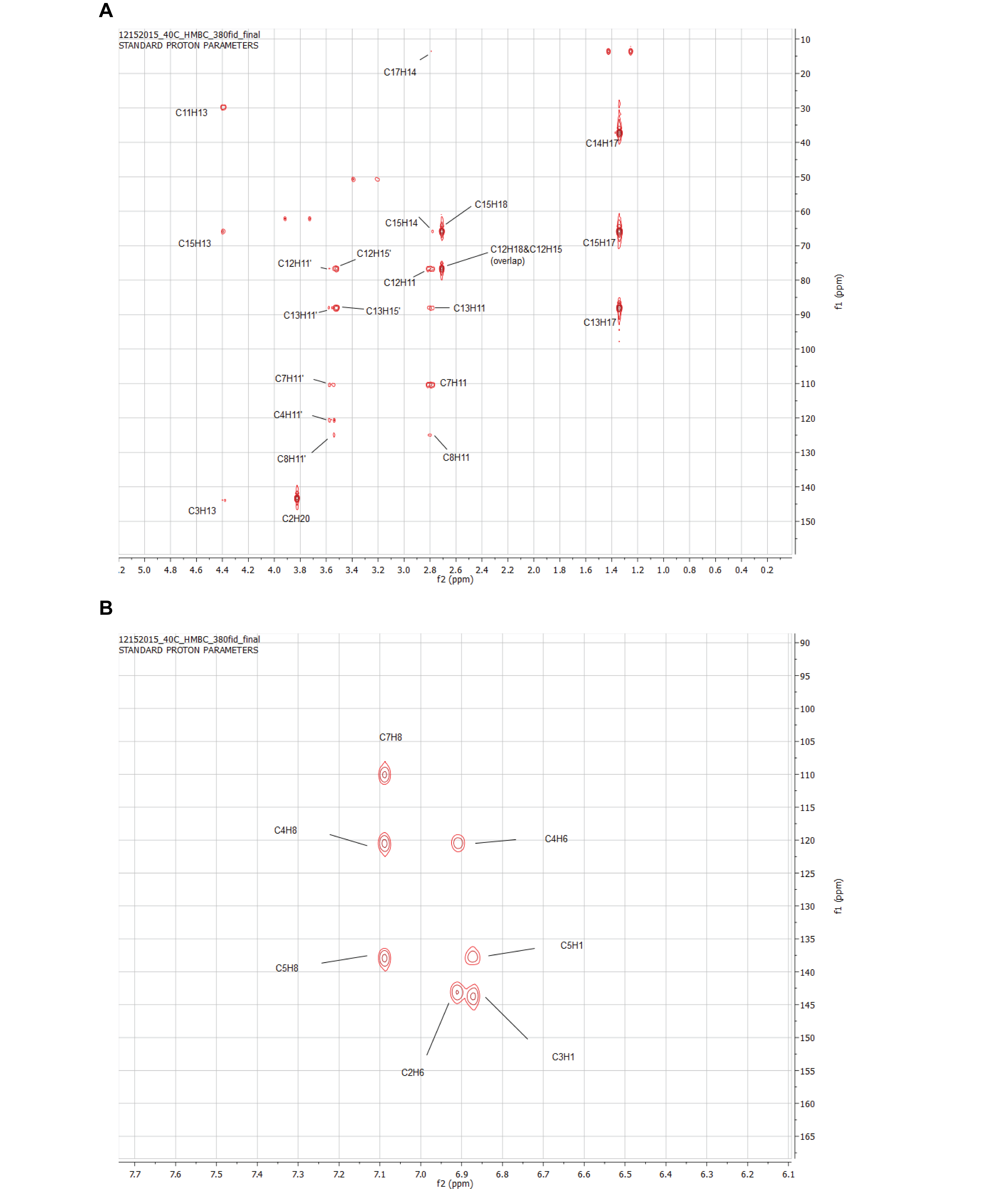

Supplement: S6 Fig — (A) Aliphatic region. Given the results from the COSY (S5 Fig) and the chemical shifts of C12 and C15 (δC 76.7 and 65.8), C12 and C15 are joined to a heteroatom. Because proton 18 (δH 2.71) has cross-peaks with both C12 and C15, it is a nitrogen atom that connects methyl group (δC 41.7, δH 2.71 on position 18), CH group (δC 76.7, δH 3.10), and CH2 group (δC 65.8, δH 2.70 and 3.52). C13 has a chemical shift of 88.2 ppm, suggesting its connection to an oxygen. With HMBC, TOCSY, HSQC, and COSY, the connectivity of the aliphatic portions is resolved. (B) Aromatic region. The connectivity-built aliphatic structure has the formula C7H13NO, which leaves C9H6NO after subtracting from the best-fitting formula. HSQC (S3 Fig) showed the existence of a methoxyl group (δC 62.2, δH 3.81). Therefore, the aromatic region was composed of C8H3N. HMBC data showed that 3 aromatic protons were located in different rings, implying a fused aromatic ring structure with one nitrogen. A substituted indole was the most common structure utilized in organisms with the matching formula. In addition, HMBC showed that protons on the methoxyl group (δH 3.81) and the aromatic proton (δH 6.90) have cross-peaks with carbon (δC 143.1), suggesting they are meta to each other. The other proton (δH 6.86) was vicinal to proton (δH 6.90) because of their coupling seen in the COSY spectrum (S5 Fig). The aromatic singlet proton δH 7.09 showed cross-peaks with 3 aromatic carbons, 2 of those carbons (δC 120.6 and δC 138.1) had cross-peaks with protons (δH 6.86 and δH 6.90), respectively, consistent with an indole configuration. HMBC further confirmed C (δC 110.6) was linked to CH2 (δH 2.79 and 3.56), and C (δC 143.7) was linked to the CH (δC 88.2, δH 4.40) across an oxygen atom. COSY, correlation spectroscopy; HMBC, heteronuclear multiple-bond correlation; HSQC, heteronuclear single quantum coherence spectroscopy; NMR, nuclear magnetic resonance; SPF, Schistosome Paralysis Factor; TOCSY, total correlation spect [file pbio.3000485.s006.tif]

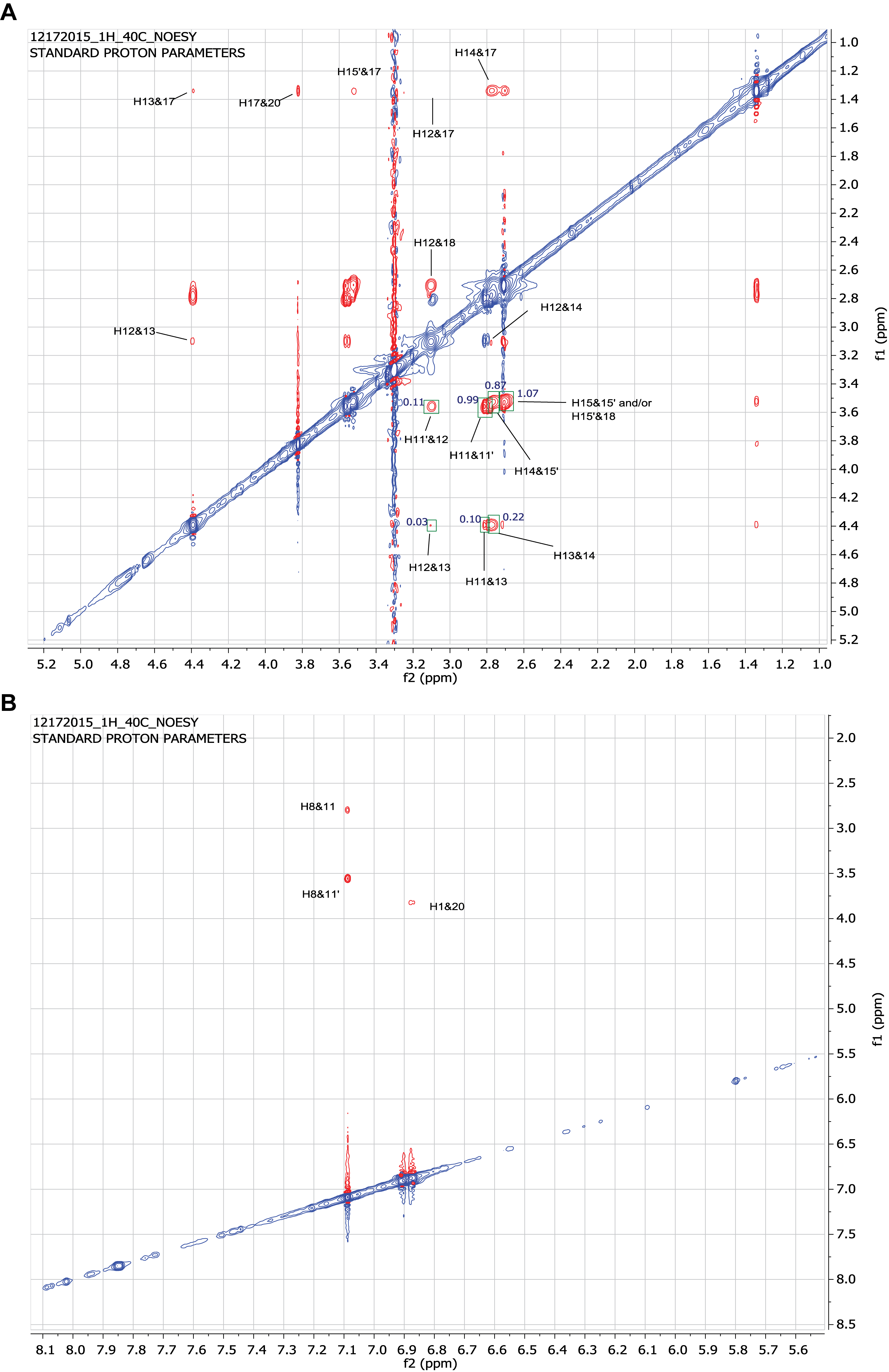

Supplement: S7 Fig — (A) Aliphatic region. The intensities of selected cross-peaks were integrated using Mnova software and shown in the spectrum. (B) Aromatic region. Results of the NOESY experiment support the final structures (Fig 2G and 2H) due to the presence of a NOE signal between H (δH 3.81) and H (δH 1.34), which could only be observed between protons with short spatial distance. For protons on the 3 consecutive chiral centers, H (δH 4.40) had an intense cross-peak with H (δH 2.77), whereas a weak signal was observed between H (δH 4.40) and H (δH 3.10) and no signal was observed between H (δH 2.77) and H (δH 3.10). This suggests that H (δH 4.40) and H (δH 2.77) are close to each other and both are distant from H (δH 3.10), which corresponds to (R, S, S) or (S, R, R) configuration on C 12, 13, 14 (δC 76.7, 88.2 and 37.2). This was further supported by NOESY signals between H (δH 2.79, 3.56) and the 3 H on chiral centers. H (δH 4.40) had a cross-peak with H (δH 2.79) but no cross-peak with H (δH 3.56). However, the opposite was observed for H (δH 3.10), which had a cross-peak with H (δH 3.56) but no cross-peak with H (δH 2.79). NMR, nuclear magnetic resonance; NOE, nuclear Overhauser effect; NOESY, nuclear Overhauser effect spectroscopy; SPF, Schistosome Paralysis Factor. (TIF) [file pbio.3000485.s007.tif]

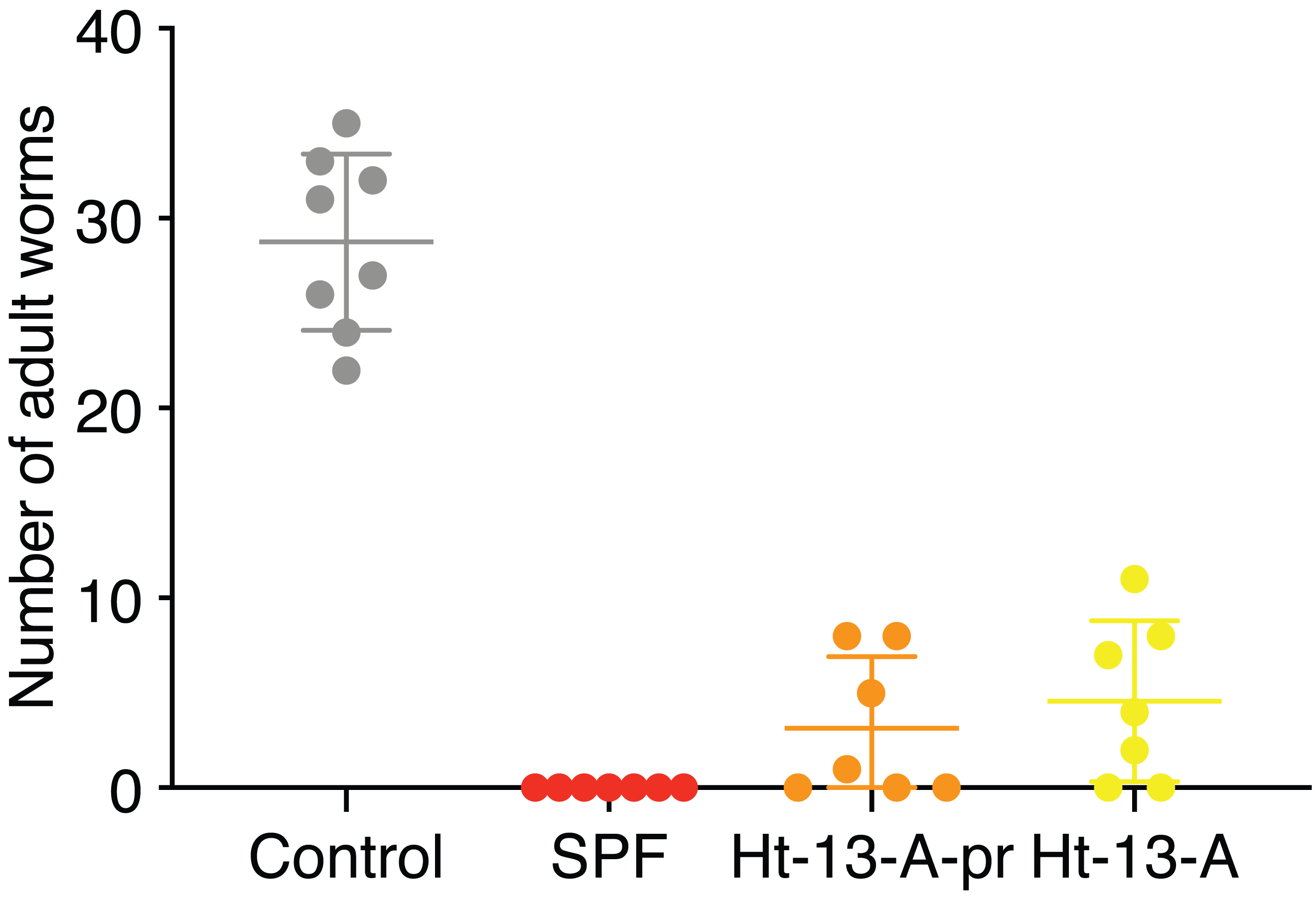

Supplement: S8 Fig — Numbers of adult worms recovered from mice exposed to approximately 100 cercariae that were pretreated with APW (N = 8), 2.5 μM SPF (N = 7), 2.5 μM Ht-13-A (N = 7), or 2.5 μM Ht-13-A-pr (N = 7). The mouse tail was lifted slightly during exposure so that its tip was 1 to 2 cm from the bottom of the test tube, avoiding direct contact with paralyzed cercariae. Data are mean ± SD, See S2 Data for corresponding raw data. APW, artificial pond water; SPF, Schistosome Paralysis Factor. (TIF) [file pbio.3000485.s008.tif]
