# Supplementary material for: A rotifer-derived paralytic compound prevents transmission of schistosomiasis to a mammalian host
Source: PLoS Biol. 2019 Oct 17;17(10):e3000485. doi: 10.1371/journal.pbio.3000485 (PMC6797223; doi:10.1371/journal.pbio.3000485)
Supplement: S1 Table — COSY, correlation spectroscopy; HMBC, heteronuclear multiple-bond correlation; HSQC, heteronuclear single quantum coherence spectroscopy; NOESY, nuclear Overhauser effect spectroscopy (PDF) [file pbio.3000485.s009.pdf]

	^{13}C			^{1}H					
Position	δ_C (detected)	δ_C (predicted)	mult.	δ_H	Peak area	mult.	COSY	HMBC	NOESY
1	116.8	110.4	CH	6.86	1.12	d (*J*=8.6 Hz)	6	3, 5	18
2	143.1	148.2	C						
3	143.7	143.1	C						
4	120.6	120.3	C						
5	138.1	132.5	C						
6	106.6	103.7	CH	6.90	1.00	d (*J*=8.6 Hz)	1	2, 4	
7	110.6	110.8	C						
8	124.9	123.0	CH	7.09	1.00	s		4, 5, 7	11,11’
9			NH						
10			O						
11	29.0	32.5	CH_2	2.79		Overlap	11’, 12	7, 8,12,13	8,11’,13
11’	29.0	32.5	CH_2	3.56	1.04	dd (*J*=14.3 Hz, 3.8Hz)	11, 12	4,7,8,12,13	8,11,12
12	76.7	72.1	CH	3.10	1.16	br	11, 11’, 13		11’,13,14,17,18
13	88.2	84.5	CH	4.40	1.05	dd (*J*=9.4 Hz, 6.8Hz)	12, 14	3,11,15	11,12,14,17
14	37.2	38.9	CH	2.77		Overlap	13, 15’, 17	15,17	12,13,15’,17
15	65.8	62.6	CH_2	2.70		Overlap	15’	12	15’,17
15’	65.8	62.6	CH_2	3.52	1.08	dd (*J*=9.6 Hz, 6.8Hz)	14	12,13	14, 15,17,18
16			N						
17	14.0	14.6	CH_3	1.34	3.01	d (*J*=6.8 Hz)	14	13, 14, 15	12,13,14,15,15’,20
18	41.7	43.7	CH_3	2.71		s		12, 15	12,15’
19			O						
20	62.2	56.1	CH_3	3.81	2.98	s		2	17
