# Supplementary material for: A rotifer-derived paralytic compound prevents transmission of schistosomiasis to a mammalian host
Source: PLoS Biol. 2019 Oct 17;17(10):e3000485. doi: 10.1371/journal.pbio.3000485 (PMC6797223; doi:10.1371/journal.pbio.3000485)
Supplement: Attachment — Submitted filename: Response_to_reviewers.docx [file pbio.3000485.s012.docx]

We thank the reviewers for their thoughtful and constructive comments. As detailed below, in this revised manuscript we have addressed all of the points that they raised. We believe that these changes have strengthened the paper and hope that it will now be suitable for publication.

*Reviewer #1*

*A few minor points:*

*1.	Lines 109-111; Please clarify. An epimer differs at one carbon but R,S suggest enantiomers. Are you just trying to say that ht-13-A and thus maybe SPF are racemic? Alternatively are you trying to say that there are R & S forms and one is responsible for the paralysis.*

Because we see biological activity in the (R,S) configurations of ht-13-A and ht-13-A-pr, but not in the epimer of Ht-13-A, we suggest that the (R,S) configuration is most likely the biologically active form of SPF. We have clarified this point in the text (new line 131) as follows:

"In contrast to ht-13-A and Ht-13-A-pr, the epimer was unable to paralyze cercariae; these results support the (*R, S*) configuration of SPF at C12, 13 as the biologically active form (Fig 2G)."

*2.	Lines 114-115; Please clarify. 30 min exposure to their tails? Did you show or just suggest that SPF works on the tails of cercariae? Did you count tails that had dislodged from cercariae? Were the heads moving on paralyzed cercariae?*

We apologize that our wording wasn't clearer. In this case, we exposed treated cercariae to mouse tails for 30 minutes to assay their ability to infect mice. SPF treatment leads to paralysis of the entire cercaria. We have clarified this point in the text (new line 140) as follows:

"We treated ~200 cercariae with different concentrations of SPF for 10 mins, and then tested their infectivity by exposing them to mouse tails for 30-min (N=6 for each condition)."

*3.	Line 147; produced by the rotifer*

This typo has been corrected.

*Reviewer #2*

*SUBSTANTIVE ISSUES*

*The observation that SPF contains a serotonin backbone comes rather “out of the blue” in the Conclusion. For this reviewer, this is one of the most important findings in the paper, being consistent with previous reports of the importance of serotonin signaling in schistosome motility and providing a jumping off point for investigations of the mode of action of SPF against schistosome cercariae. Given this, shouldn’t this point be introduced in Results and Discussion? At or around line 90, where the possible structures of SPF are introduced would seem to be a logical point for this introduction. It might also be helpful to shade the serotonin backbone in the structures of SPF, ht-13-A, ht-13-A pr and ht-13-B in Fig. 3.*

We thank the reviewer for this excellent suggestion and have moved the first mention of the similarity to serotonin earlier in the Results section. Rather than line 90, as suggested by the Reviewer, we felt it was more appropriate to mention serotonin in the context of the two chemical analogues, ht-13-A and ht-13-B, which were first isolated based upon their binding to serotonin receptors, providing functionally relevance to the similar chemical structures. As suggested, we have also shaded in the serotonin backbone of SPF in Fig. 3A. We have decided not to shade in the serotonin backbone in the analogues because it would be redundant and it would also distract the reader from seeing the chemical differences between the analogues. We hope that this reasoning will be acceptable. The text has been revised as follows (new line 119):

"Two natural compounds from *Streptomyces sp.*, ht-13-A and ht-13-B, are structurally related to SPF; they were isolated based on their affinities for human serotonin receptors (17). All three alkaloids share a novel oxepineindole framework fused with a pyrrolidine ring (Fig 3A-C; note the serotonin backbone highlighted in red in Fig 3A)."

*MINOR ISSUES OF COPY EDITING AND CLARITY*

*Line 147: Insert “by” between “produced” and “the rotifer”.*

*Line 162: Insert “a” before “100 um cell strainer”.*

*Line 205: Suggest substituting “using a” for “through”.*

*Lines 254-255: I assume the Tricaine incubation was to unpair the adult worms for counting, correct? If so, I suggest “…recovered by hepatic portal vein perfusion, and males and females were unpaired by a brief incubation in 2.5% Tricaine to facilitate counting.”*

All of these edits and suggestions have been incorporated.

*Reviewer #3*

*I recommend publication with no changes.*

*Reviewer #4*

*I thought the paper was interesting, though the biosynthetic origin of the tetracyclic alkaloid (called Schistosome Paralysis Factor, SPF) should really be discussed in the manuscript. Other than SPF, the Streptomyces-derived alkaloids ht-13-A and ht-13-B are the only two examples of naturally occurring 3,4-oxepino-fused indoles, and thus it seems likely that the SPF is also bacterial origin, rather than “produced by the rotifer Rotaria rotatoria”, as the abstract suggests.*

*Some experiments to explore a potential bacterial origin of SPF would be in order. Did the authors check for the presence of any Streptomyces spp in Rotaria rotatoria? Sequencing would provide a quick means to get an overview of associated microbiota.*

This is a great idea and represents an important next step in the work, but we would suggest that this is well beyond the scope of the current manuscript, and best left to future work.

For the requested experiment to be informative, we will need to characterize the microbiomes of *R. rotatoria* (which makes the paralytic compound) and *P. acuticornis* (which does not). We grow both species under identical culture conditions, feeding them a commercial rotifer food, which contains phytoplankton, yeast, vitamins, minerals, and presumably, whatever bacteria can also grow when this concoction is added to the culture water. Because rotifers feed on microorganisms in their water, we have every reason to believe that we will find lots of bacteria associated with both *R. rotatoria* and *P. acuticornis* (as well as their food source). Given how common *Streptomyces sp.* are, it seems likely that we will find *Streptomyces* associated with both rotifers.

Crucially, even if we find some *Streptomyces sp*. associated specifically with *R. rotatoria*, such a result will by no means provide evidence that it is the source of SPF. Although the two known chemical compounds related to SPF are produced by *Streptomyces*, this does not mean that a related compound must have a bacterial origin. This is particularly true because horizontal gene transfer is well documented in rotifers. It seems equally plausible that this rotifer could have incorporated bacterial genes encoding SPF biosynthesis pathway components.

For these reasons the suggested experiment seems roughly equivalent to another thesis project, rather than an additional piece of data necessary to bolster the conclusions of the current paper. To clarify this issue, we have expanded our discussion of this issue (line 182):

"Because compounds with structural similarities to SPF are produced by *Streptomyces sp*., it will be important to examine the possibility that SPF is not directly produced by the rotifer, but rather by constituent(s) of its own microbiome. However, given that horizontal gene transfer is well documented in rotifers (31, 32), it is also possible that *R. rotatoria* has acquired the synthetic machinery to produce SPF on its own. Future work will help reveal the source of SPF and its biosynthetic pathway."
